# Leading online learning during a pandemic and beyond: Challenges and opportunities for school leaders in England

**DOI:** 10.1177/17411432231191171

**Published:** 2023-08-23

**Authors:** Alan Floyd, Jacqueline Baxter, Andres Morales, Rehana Bari

**Keywords:** Strategic leading of online learning, professional learning and knowledge, collaborative professional practice, innovation

## Abstract

Emerging work around the globe has identified the impact of closing schools and moving education online during the COVID-19 pandemic has had, especially on children from disadvantaged backgrounds. Whilst research into this area is growing from both students’ and parents’ perspectives, there remains a need to explore how school leaders strategically dealt with these challenging circumstances and, crucially, what can be learned for the future to ensure some of the opportunities that emerged can be harnessed and developed. This article addresses this knowledge gap by drawing on an UKRI-funded mixed-methods study involving an online survey (*n* = 65) and semi-structured interviews (*n* = 50) with headteachers in England exploring not only the strategic challenges that school leaders had to overcome but also highlighting the opportunities that have emerged from the crisis that will positively impact on leading online learning in the future. The results from this research have been used to develop a free online professional development course for heads, underpinned by the key findings, which are highlighted at the end of this article.

## Introduction

Throughout the COVID-19 pandemic, schools have had to rapidly react to changing learning strategies brought about by worldwide governments introducing a variety of restrictions to try to control the spread of the virus. In the UK, this has meant extended periods of school closures (or ‘lockdowns’) where the majority of children were forced to stay away from school and learning was moved ‘online’. Emerging work around the globe has identified the huge negative impact these educational policies have had, particularly in the developing countries of the Global South (Blackman, 2022), and on students from disadvantaged schools or disadvantaged backgrounds more generally (OECD, 2021), with calls for more work to be done to explore the impact on children with special educational needs (Bond, 2020). Whilst research into this area is growing from both students’ and parents’ perspectives (see, e.g., Dong et al., 2020; Khlaif et al., 2021; Misirli and Ergulec, 2021), there remains a need to explore how school leaders strategically dealt with these challenging circumstances and, crucially, what can be learned for the future to ensure some of the opportunities that emerged can be harnessed and developed. In work published elsewhere, which is also based on the research project reported in this article, we explored digital strategy in English secondary schools during this period and argued that,

…in order for leaders to progress to a whole school digital strategy, the learning during and post pandemic must be harvested in such a way that it can be used to support digital strategies more effectively, or the creativity and learning that have occurred will be lost in the pell mell of school life. (Baxter et al., 2023: 17)

Thus, this article addresses this knowledge gap by extending this previous work and exploring not only the strategic challenges that school leaders had to overcome, including dealing with the impact of the restrictions on children with differing educational needs, such as special educational needs and disabilities (SEND), those who have English as an additional language (EAL), and those on free school meals (FSM), but also highlighting the opportunities that have emerged from the crisis that will positively impact on leading online learning moving forward. As such, it offers a significant and original contribution to knowledge with clear practical implications for improved policy and practice in this area.

## Conceptual framework

Since 2000, several frameworks have been developed and used to evaluate digital learning in the UK and elsewhere, each highlighting key aspects of both staff competencies and the organisational climate needed for successful online learning development. In our previous work (see Baxter et al., 2023), we draw on a number of these frameworks to investigate key components of effective digital learning. These include the Community of Inquiry (CoI) framework developed in 2000 which assesses cognitive presence, social presence and teaching presence (Garrison et al., 2000); the Technological Pedagogical Content Knowledge (TPCK) framework, introduced in 2006, which considers the intersection of technology, pedagogy and content knowledge (Mishra and Koehler, 2006); Eickelmann's (2011) ‘supportive and hindering factors’ for sustainable digital implementation in schools; and Puentedura's SAMR model which categorises technology integration into substitution, augmentation, modification and redefinition (SAMR) (Puentedura, 2014). A more recent framework, the Digital Competence Framework, was released in 2019 by the European Commission as part of their ‘key competencies for lifelong learning’ report, offering guidelines for effective digital learning implementation (European Commission, 2019). These frameworks contribute to evaluating and enhancing digital learning environments, and alongside strong leadership, key factors emerge viewed as essential to the delivery of digital learning which include staff knowledge and competency, collaboration and sharing ideas internally and externally, ongoing staff development and learning and freedom and encouragement for staff to innovate and look for creative solutions. Thus, in this article, we have chosen to focus on exploring the challenges and opportunities that school leaders experienced during this time by merging these factors into three overarching and interconnected concepts, namely professional learning and knowledge, collaborative professional practice and innovation. Through exploring existing and required professional learning and knowledge, we can explore whether school leaders felt that they and their staff had the necessary skills and experience needed to adapt to the challenges of school closures and the rapid move to online learning. To understand this issue in more depth, it is necessary to examine how collaboration was used by school leaders and staff to share knowledge and improve learning throughout the school community, whilst simultaneously highlighting the key aspects of innovation that allowed schools to be successful in delivering crucial student learning aims through such challenging circumstances. We argue here that using this framework aligns our focus to relationships, challenges and opportunities experienced throughout the whole school community during this period and, as part of this study, look to reflect on its efficacy in the concluding section of the paper. The following discussion develops this thinking further.

In relation to professional learning and knowledge, previous work on digital learning suggests that staff knowledge and expertise is one of the key challenges to implementing a successful digital learning strategy and that it is crucial to move the focus from the mere presence of ICT provision in schools to ensuring its impact on knowledge acquisition (Eickelmann, 2011). Hence, successful staff professional learning processes are key for transforming conventional teaching into new online learning models (Napal et al., 2020; Vermeulen et al., 2015). This issue is exacerbated when dealing with the knowledge and skills needed to support children with differing educational needs such as SEND and EAL and those on FSM, which need greater understanding and research focus (Bond, 2020).

A recent report published by the Organisation for Economic Cooperation and Development (OECD) suggested that, from PISA data gathered in 2018, on average across OECD countries, only 65% of 15-year-olds were enrolled in schools in which the headteacher perceived their staff to have the necessary technological and pedagogical skills to implement online learning successfully. However, from their rapid survey of 330 education leaders representing 98 different countries at the start of the pandemic, they found that the development of massive open online courses (MOOCs) or intensive e-training for teachers dedicated to the creation of distance learning courses were two of the most successful strategies used by the school leaders (in some cases supported by the government) to enhance the teachers’ professional skills. These findings suggest that by providing an appropriate e-learning environment for staff (i.e. participate in MOOCs or engage in online communities to share resources and experiences with other teachers), the delivery of online learning during the closures and restrictions was enhanced, even if some professionals did not have prior experience in this area (Reimers and Schleicher, 2020).

Linked to professional learning is the notion of collaborative professional practice both within the school and with external stakeholders such as parents and other community members. It is clear that working collaboratively and sharing knowledge and expertise provides more opportunities for growth, as highlighted in a number of studies on school effectiveness and trust (see Ehren and Baxter, 2020). This is especially true in relation to leading online learning provision where headteachers and teaching staff may not have the skill set and experience necessary to flex and adapt to new technological innovations in a very quick changing field. As an example, a study conducted by Sheffield et al. (2018) in Australia explored the key components of highly effective teacher professional learning to leverage transformational change in relation to online learning: 28 primary and secondary school teachers collaborated through a guided Professional Learning Network to develop and reflect upon their digital capabilities. The findings from this study show that a long-term, embedded professional learning programme can be successful in improving the confidence and preparedness of teachers in implementing a mandated curriculum change in relation to online learning. A similar study conducted in New Zealand also highlighted the importance of collaborative approaches for staff development linked to online learning, particularly the use of social media to encourage more collaboration amongst professionals (Mostafa, 2021). Finally, Lantz-Andersson et al. (2018) undertook a systematic review of 52 empirical studies of formally organised and informally developed online teacher communities from the early 2000s to 2018. This desk research focused on the social as well as technological aspects of online participation and illustrates how teacher learning communities are shaped by broader contexts of relationships and belonging. These findings reveal that whilst formally organised and informally developed communities address different needs amongst teachers and supported different outcomes, online communities can be a valuable means of developing supportive and collegial professional practices.

The final interconnected concept in our framework is innovation. Previous research into the pandemic has shown that innovation can flourish, with people successfully overcoming online learning challenges even in the face of great adversity (Gomersall and Floyd, 2022). Within the context of this study, digital innovation is of particular interest to school leaders when having to move all teaching and learning online almost overnight (Dwivedi et al., 2020). This reality posed unprecedented challenges for students, who needed technical assistance, but also for staff and school leaders, who had to reinvent themselves in record time to keep schools operations running. Such a rapid shift to online education requires appropriate infrastructure and technological platforms, solid servers that can sustain the virtual workload and methodological training of teachers and students for online delivery using all the technical and educational resources available (Oke and Fernandes, 2020). Many schools have signed contracts with companies such as Microsoft that provide Office or Teams resources or technological platforms to strengthen virtual communication, and a wide variety of online communication platforms and solutions, considered as innovative in education, have been used such as instant messaging tools (e.g. WhatsApp, Telegram), video-conferencing tools (e.g. Zoom, Teams, Skype, Google Hangouts, Google Meet) and educational apps (e.g. Google Classroom), combined with email and telephone conversations to maintain individualised contact with students to help digitalise the entire teaching–learning process (Babinčáková and Bernard, 2020; Tamah et al., 2020). This article aims to explore English headteachers’ perceptions of these experiences, as well as identify aspects of pedagogical innovation that can help schools move forward successfully with online learning in the future. Therefore, the main research question addressed is:

- What are school leaders’ perspectives and experiences of the challenges and opportunities of leading online learning during COVID-19 restrictions and beyond, and what lessons can be learned for the future?

## Methods

### Design

To address our research question, the study used a two-stage, sequential explanatory mixed-methods approach (Creswell, 2014) involving an initial online survey (*n* = 65) followed by semi-structured interviews with school headteachers and CEOs of MATs (*n* = 50). Originally, it was hoped to gain survey data from a larger sample size, but poor uptake even after incentivisation meant that we subsequently abandoned this plan and used the survey data to identify emerging themes to explore in more detail in the semi-structured interviews. Ethics permissions were obtained from all participating universities, in line with BERA protocols, which included information about the project and signed consent forms for interview participants and full ethical disclosure at the beginning of the survey. All school names and data have been anonymised throughout.

### Survey

The survey was developed in line with published guidelines to ensure validity in questionnaire design (see Gehlbach and Brinkworth, 2011). At the outset, a draft survey was created based on themes that emerged from an initial literature review and 10 subsequent semi-structured pilot interviews with headteachers. This draft survey included questions related to the following themes: in what way online learning was part of the school curriculum before, during and after COVID restrictions; what the key challenges and opportunities were for school leaders during this time period; what support and staff learning needs there were; how leaders perceived that the restrictions impacted children with various learning needs; what engagement and collaboration had been undertaken and how digital learning might be used/developed by school leaders in the future. This draft was subject to an initial peer review, followed by a full validation and piloting exercise. First, the survey link was sent to our advisers and project partners who were asked to complete the survey and provide feedback on timing, item clarity, appropriateness and coverage of issues pertinent to the aims of the project. We then held a focus group meeting with this group to discuss each of the questions in turn. Several changes to wording were made following this exercise. The final draft survey was then piloted with 10 headteachers. The survey was reviewed by the Open University and University of Reading Ethics Committees and given a favourable ethical opinion for conduct. It was designed to be completed within 10 min to ensure that participants could engage fully with the process without an unduly lengthy time commitment. At the end of the survey, participants could volunteer to put themselves forward for a follow-up virtual face-to-face interview.

Following a front page which included key ethical information and details about the research project, the final survey contained 34 questions over five sections. The first section (About You) contained demographic questions about the individual completing the survey and how long they had been in the role. The second section (About Your School) contained questions related to school type, size, whether the school was part of a Multi Academy Trust (MAT) and its subsequent size, and key information linked to the percentage of pupils on roll with SEND, on FSM and who have EAL.

The rest of the survey was split into three sections:

- Questions about online learning strategies and practices before COVID-19 restrictions were introduced.
- Questions about online learning strategies and practices during COVID-19 restrictions.
- Questions about proposed online learning strategies and practices after COVID-19 restrictions are lifted.

Each of these sections contained a range of closed questions where participants ticked a relevant box in response to a particular question, for example, *In what way was online learning part of your curriculum?*; ranking questions where participants were asked to rank certain aspects of leading inline learning, for example, *Please rank the following challenges of leading online learning before COVID-19 restrictions were introduced in order of challenge – 1 being the most challenging, 5 being the least*; and Likert scale questions where participants were asked to agree or disagree with certain statements, for example, *How much do you agree or disagree with the following statements before COVID-19 restrictions were introduced?* with each answer based on a four-point agreement scale ranging from strongly agree to strongly disagree. Each section of the questionnaire also had an open comments section for participants to add qualitative comments about their responses if appropriate, for example, *Do you have any other comments about leading online learning during COVID-19 restrictions?* The survey was distributed to headteachers via a range of routes:

- Direct email via partner databases and networks
- Social media platforms, e.g. Twitter
- Snowball sampling (passed on from participant to participant)

As previously discussed, uptake was very low (*n* = 65) no doubt in relation to the bombardment of policies that headteachers were facing via email on a daily basis during this period (as identified in our semi-structured interview data). Table 1 shows the different school types represented in the survey data. Of these, 33 schools (55.9%) were part of a MAT and size of school varied from 18 (28.6%) that were below 500 pupils, 23 (36.5%) that were between 501 and 1000 pupils, 16 (25.4%) between 1001 and 1500, and 6 (9.5%) that had over 1500 pupils on roll.

**Table 1.** Survey School Type

School Type	Number	% of total
Academy	15	23.1
Free school	10	15.4
Maintained/community school	8	12.3
University Technical College	7	10.8
Faith school	6	9.2
Further Education College	5	7.7
Grammar school	3	4.6
Other (e.g. special school, PRU)	8	12.2

Notes: Percentage totals may not equal 100 due to rounding

The breakdown of pupils in relation to SEND, FSM and EAL is given in Table 2. For context, national average percentages are as follows: SEND – 12.9%; FSM – 17.3%; EAL – 17.1%.

**Table 2.** Breakdown of pupil numbers in relation to SEND, FSM, and EAL.

Percentage of pupils	SEND	FSM	EAL
0-5%	9 (14.5%)	7 (11.3%)	15 (25%)
6-10%	23 (37.1%)	13 (21%)	14 (23.3%)
11-15%	**14 (22.6%)**	**14 (22.6%)**	8 (13.3%)
16-20%	9 (14.5%)	14 (22.6%)	**10 (16.7%)**
Above 20%	7 (113%)	14 (22.6%)	13 (21.7%)

Notes: National average band in bold

### Interviews

Following a pilot study, the main interviews were carried out online using Microsoft Teams with each one lasting between 1 and 1.5 h. The sample consisted of heads of single schools and CEOs of MATs as shown in Table 3. These academies are run by not-for-profit trusts rather than Local Authorities and held to account by contracts held with central government rather than by the traditional model of school governing bodies. Approximately 40% of the schools were located in areas of low social economic status chosen due to their above average number of students receiving FSM. Participants were recruited through our three school support project partners (Schools North East, Derbyshire Teaching Alliance and The Key for school leaders) and direct approaches via social media. The interview schedule was developed using themes that emerged from an initial literature review and from the survey data analysis. These were then peer-reviewed by our project partners (all senior educational leaders or researchers) and piloted. The interview schedule is included in the Appendix, although questions were not necessarily asked in order, to allow the conversation to flow more easily.

**Table 3.** Interview Sample

Type of Organisation	Role	Number
Stand Alone Academy	Headteacher	20
Local Authority School	Headteacher	22
MAT	CEO	4
Community School	Headteacher	2
Special Schools	Headteacher	2

### Analysis

The survey data were analysed initially using descriptive statistics. Cross-tabulations and non-parametric tests were then undertaken to see if there was a change of engagement with online learning provision before and during COVID-19 restrictions, and whether there was an association between online learning provision before COVID-19 and certain key measures (*P* < 0.05). These non-parametric tests allowed us to explore whether our distribution of frequencies were significantly different from those expected by chance alone (Nardi, 2018).

The interviews were analysed following thematic analysis techniques outlined by Lichtman (2013) and suggestions for ensuring rigour in qualitative analysis described by Creswell (2014) and managed using Nvivo qualitative data analysis software: two researchers coded each transcript separately using a code book derived from the pilot study (for more details of this process, see Baxter et al., 2023) and then grouped these codes together to form initial themes. The researchers then discussed and refined these emerging themes, thus allowing for inter-researcher agreement, and then related them to the study's conceptual framework to develop theoretical conclusions. See Table 4 for an example of this process.

**Table 4.** Example of thematic analysis

Initial Codes		Themes/Categories		Concepts
Staff stepping up Teacher creativity Doing remarkable things Adapting Shifting culture	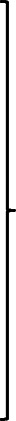	Institutional growth Sense of creativity	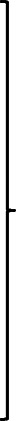	*Opportunities* Pedagogical Innovation

Both sets of results were then combined and are presented thematically below.

## Findings

### Prior knowledge and experience

Before COVID-19 restrictions were introduced, data from our survey suggests that online learning was part of the school curriculum (including homework tasks) for 41 of the schools (66.1%) in contrast to 21 (33.9%) that did not have any online learning provision. Of the 41 schools that used online learning, only 14 (34.1%) stated that online learning was fully embedded within the whole curriculum, with 21 (51.2%) headteachers stating that online learning was used for certain subjects and activities (in school and homework tasks), and 6 (14.6%) reporting that online learning was only used for homework tasks. This finding has clear implications for the ability for schools to experience a successful transition to full online learning when restrictions and lockdowns were introduced at the start of the pandemic.

In fact, when asked to rank the main challenges of leading online learning *before restrictions were introduced*, a lack of staff expertise was identified as either the first or second main challenge for the majority of school leaders (*n* = 37; 69.8%), with a lack of pupil expertise identified as the main or second ranked key challenge for 28 (52.8%), and a lack of equipment or available home provision for pupils being identified as the main or second ranked key challenge for 23 (41.8%). As one headteacher put in the open text box:

This type of learning was not even considered prior to Covid-19

and another stating:

Online learning was not in place prior to Covid-19. As a free school that started in 2017 this was not something that we had put any time in to preparing for at this stage.

These findings were also reflected in the interview data. Almost all participants recognised that they did not have either experience or appropriate knowledge to implement online learning in their schools. For example, one headteacher explained that before the restrictions:

… we did very little online learning really. We started to investigate using teams, but it was more of a kind of a platform to store resources and for students to be able to access rather than actually delivering anything online at all. This was across the curriculum. Pre-covid we had explored things like Google Docs and using different platforms to communicate with kids in a different way.

This lack of knowledge and experience created major problems for some schools as the following participant highlighted:

A key challenge was not having the knowledge of knowing the online ecosystem. It was uncomfortable for everyone not having the comfort of knowing and not having all the answers … we had to let go of perfectionism, which is not part of our culture … and I was telling staff that we can only all do our best at the time

### Staff development needs

From the survey, 35 school leaders (56.5%) indicated that the person responsible for online learning had changed since the introduction of COVID-19 restrictions. The breakdown of who is now responsible is shown in Table 5 and suggests that many schools have distributed this responsibility across several key staff within their schools. Whilst this finding shows flexibility and collaboration in working practices during the crisis, it has implications for staff development and training for these schools moving forward which will be discussed later.

**Table 5.** Who is now responsible for online learning during COVID-19 restrictions?

Person responsible	Number	% of total
Headteacher	10	15.4
Deputy head	9	13.8
Assistant head	17	26.2
Key stage leaders	8	12.3
Department heads/subject leads	14	21.6
Individual teachers	6	9.2
Other	1	1.5

Note: Percentage totals may not equal 100 due to rounding.

Indeed, when asked to rank the main challenges of leading online learning during COVID-19 restrictions, a lack of staff expertise was again identified as either the number one or second main challenge for 31 of the school leaders (56.4%), with a lack of pupil expertise identified as the main or second ranked key challenge for 22 (41.5%), and a lack of equipment or available home provision for pupils being identified as the main or second ranked key challenge for 32 (55.2%). Interestingly, behaviour management was only identified as a main or second ranked key challenge for 13 schools (24.1%), with 21 schools (38.9%) ranking this as the least challenging aspect of leading online learning during the restrictions and 7 (13.0%) stating that it was not an issue at all.

When asked to rank the support and guidance they have received for leading online learning during COVID-19 restrictions, peer support and existing staff expertise were identified as either ‘very useful’ or ‘moderately useful’ for both the school leaders whether they had online learning provision before COVID-19 restriction or not. The schools that did not have any online provision before COVID-19 restrictions were identified ‘other’ such as Webinars, Edu social media, ASCL and ‘external consultancy’ as more useful than the schools that did have online provision before COVID-19 restrictions. Both school leaders ranked ‘parental expertise’ as least useful.

Table 6 shows findings from the survey as to what support and guidance headteachers felt would have helped them during COVID-19 restrictions, which included software support and guidance (*n* = 36; 58.1%), curriculum support (*n* = 33; 53.2%) and staff development and guidance (*n* = 33; 53.2%). These findings all link to staff development needs and will be discussed in more detail later in this article.

**Table 6.** What support and guidance would have helped during the restrictions?

Type of support and guidance	Number	% of total
Software support and guidance	36	58.1
Curriculum support and guidance	33	53.2
Staff development support and guidance	33	53.2
Hardware support and guidance	31	50
Peer support (from other school leaders)	16	25.8
Other	1	1.6

Note: Multi-answer: percentage of respondents who selected each answer option (e.g. 100% would represent that all this question's respondents chose that option).

Almost half the headteachers surveyed (*n* = 31; 50%) reported that they wanted more help and guidance in relation to hardware. Concerning laptop/hardware provision from the government, 45 (71.4%) heads requested support with 34 of these schools (77.3%) receiving equipment and 10 schools (22.7%) disappointingly not receiving any of the hardware support they requested. One head gave more details of some of the challenges of gaining support in this area in the open comments box:

We received a total of 61 devices, 56 in January 2021 then the remaining 5 in March 2021. More than 96 families had barriers to access – this helped to an extent but was not sufficient. We are still awaiting 4G routers from DfE, although we managed to source some from another school.

From the interview data, several key challenges were identified with ‘staff training’ being the phrase most mentioned in the transcripts when talking about needs. Other challenges were linked to accessing appropriate equipment and the impact on staff in relation to welfare as the following comments show:

By far access to kit was the biggest challenge. Sourcing kit, getting build and virus software installed and everything else that was needed and then delivering. Our computing lead was taking the mini bus out and literally had the van full of kit. It was a really demanding time. I think the lack of support from the government, their communications were vague at best and no advice on how to actually implement what they were asking.

… but there's been a big emotional impact from 2020, all my staff have been personally impacted. Their wellbeing has been affected and they are exhausted. There's an element of trauma in my workforce and in the community.

#### Impact on pupils with SEND, with EAL or on FSM

In the survey, school leaders were asked whether pupils with SEND, with EAL, and on FSM were able to engage fully with online learning provision during COVID-19 restrictions and the results of these questions are shown in Table 7. It is clear from these results that whilst the majority of headteachers felt that pupils in these groups were able to engage fully, there were still a worrying number of school leaders who felt that they had not been able to: 10 (16.7%) for pupils with SEND, 21 (34.4%) for pupils with EAL, and 11 (18.7%) for pupils on FSMs. These results confirm that COVID-19 restrictions appear to disproportionally negatively affect pupils in these groups in relation to engaging with online learning.

**Table 7.** Impact on pupils with SEND, with EAL or on FSM.

Answer options	*n*	%
*Pupils with SEND were able to fully engage with online learning provision*		
Strongly agree	14	23.3
Agree	36	60
Disagree	10	16.7
Strongly disagree	0	0.0
*Pupils with EAL were able to fully engage with online learning provision*		
Strongly agree	12	19.7
Agree	28	45.9
Disagree	20	32.8
Strongly disagree	1	1.6
*Pupils on FSM were able to fully engage with online learning provision*		
Strongly agree	13	22.0
Agree	35	59.3
Disagree	8	13.6
Strongly disagree	3	5.1

Note: Percentage totals may not equal 100% because of rounding.

The interview data also highlighted the fact that lockdowns had impacted most heavily on those children from more deprived backgrounds:

50% of our children are from lower classes with lower prior attainment and don’t have that access. So, that has been a real driver for us. Considering how we adapt our curriculum so that it is sharp and it is progressive. I am genuinely concerned about the breadth of curriculum and opportunity that children would have lost, especially those who are from deprived backgrounds …

During the first lockdown it was relentless in making sure we were meeting children's needs at home, as well as, those children in school, which was SEND pupils and those of key workers … For those at home, the emphasis was on safeguarding and making sure our children's’ needs were met. We couldn’t deliver the same level of learning. It was relentless in printing packs and delivering worksheets so children had something to do as they didn’t have access to being online and weren’t set up to have anything online. We created food hampers, stationery packs, study packs …

### Stakeholder engagement

Encouragingly from the survey, 50 schools (80.6%) engaged with parents/carers/guardians in developing their online learning provision, the details of which are shown in Table 9. Even more encouragingly, 51 schools (83.6%) engaged with pupils when developing their online learning provision, the details of which are also shown in Table 8. These results suggest a more collaborative model of teaching and learning development than perhaps existed before restrictions were introduced.

**Table 8.** Stakeholder engagement.

Answer options	Number	%
*Details of stakeholder engagement – parents/carers/guardians*		
Survey	32	64.0
Email	28	56.0
Phone calls	28	56.0
Social media platforms	27	54.0
Parent–teacher association communication	9	18.0
Set up special ‘COVID-19’ parent–teacher committee	8	16.0
*Details of stakeholder engagement – pupils*		
Survey	35	68.6
Social media	27	52.9
Email	26	51
Phone calls	24	47.1
Set up special ‘COVID-19’ pupil–teacher committee	7	13.7
Other	3	5.9

Note: Multi-answer: percentage of respondents who selected each answer option (e.g. 100% would represent that all this question's respondents chose that option).

**Table 9.** Online learning provision implemented during the restrictions.

Online learning provision implemented	Number	%
Developed remote synchronous learning activities (e.g. ‘live’ face-to-face sessions using online video or learning platform)	52	82.5
Set up remote ‘face-to-face’ tutoring sessions (using online video platform)	44	69.8
Developed in-person synchronous learning activities for pupils in school (e.g. using laptops, tablets or phones for learning activities in class)	40	63.5
Developed asynchronous learning activities (e.g. online worksheets to be completed by students in their own time)	29	46
Run whole school assemblies online	27	42.9
Other	4	6.3

Note: Multi-answer: percentage of respondents who selected each answer option (e.g. 100% would represent that all this question's respondents chose that option).

From the interviews, engagement had been linked to asking pupils about their online interactions and what help they were getting at home, as the following example shows:

We tried also to access parents, but we found that parent engagement was very, very low for us. So in the survey of the children, on average a third of our children got any form of help from their parents and carers at home. So 70% of our children were working independently … and that did account for some of the lower levels of engagement, so we did a lot of work to re-engage those children. We did weekly phone calls where we could.

Some headteachers pointed out how using online analytics had helped them identify children who were staying up very late to complete their work:

Around safeguarding, we actually audited that there were kids on-line at two in the morning, and we raised that as flags with their parents, that they were engaging in school work at those times … and we didn’t feel that was healthy or right.

And others spoke of how they wanted to set up more training and support for parents, which in turn would enable more children to engage more positively with online learning, especially those from disadvantaged backgrounds:

I would like to have done more training of the parents. So when we move forward, and if we ever get back to normal. my disadvantage strategy will be getting the parents of the disadvantaged children in Year 7. Teaching them how to use the machines and giving the kids a computer and ensure they’ve got Wi-Fi. I’ve seen the amount of learning my own son does independently on his computer. I just feel angry with myself if you like that I didn’t pick that up as an easy solution.

As well as engagement with pupils and parents/carers, our analysis of the interview data suggested that there was increased stakeholder engagement and collaboration across schools within MATs, as the following example highlights:

It was about collaboration across the trust and sharing resources. And that was the driver for it, because the trust is from School A, which is on the edge of the town, which is our eastern most school, over to School B, which is an hour and a half drive. So, School B is a bit of an outlier for us. The other schools are all quite close. So, although we do move between … some staff move between schools in the trust, it became obvious that we needed a more efficient way of sharing resources and having common work areas.

### Innovation

Table 9 shows activities that schools have implemented during the restrictions. ‘Other’ activities included survey data highlighting the range of different online remote pastoral and SEND engagement, extra-curricular activities, and house events and competitions.

A total of 56 school leaders (86.2%) made key changes to the management of online learning in the period between the first lockdown in March 2020 and the second principal lockdown in the winter of 2020/2021, which included developing more suitable online learning activities and ensuring that learners were able to engage more fully with the curriculum as shown in Table 10. These data highlights some of the institutional growth and development that has emerged in response to the crisis, showing evidence of the pedagogical innovation that has arisen in an attempt to improve online teaching and learning provision for pupils during the restrictions.

**Table 10.** Change in online provision from the first lockdown to the second.

Answers	Number	%
Developed more synchronous (real time) activities	41	65.1
Run more face-to-face tutorials (using online video platforms)	35	55.6
Developed more asynchronous (accessed anytime) activities	31	49.2
Run more whole school assemblies	22	34.9
There has been no change to our online provision; we were happy with how it went the first time	9	14.3
Other	1	1.6

Note: Multi-answer: percentage of respondents who selected each answer option (e.g. 100% would represent that all this question's respondents chose that option).

Such innovation was also very evident from the interview data, as shown in the following quote:

When you’ve got five science departments and five PE departments all working together and meeting together and sharing ideas, that's been very powerful. And our most creative and engaged staff have made full use of the networks on Twitter and everything else that exists now. And there is a real sense of creativity and support I think across the profession. The staff have done a lot of collaborative enquiry and staff have been sharing insights on online learning …

### Imagined future possibilities

In the survey, when asked what role they thought online learning will play after COVID-19 restrictions are lifted, 27 leaders (42.9%) thought that it would play a major role, 31 (49.2%) thought it would play a medium role and only 5 (7.9%) felt that it would play a minor or no role. Table 11 shows the different strategies that school leaders are putting in place for online learning in the future, with 29 (46.0%) fully embedding online learning within the whole curriculum.

**Table 11.** Future strategies for online learning in your school.

Answer options	Number	%
*Strategies*		
Online learning will be embedded within the whole curriculum (including homework tasks)	29	46.0
Online learning will be used for certain subjects and activities (in school and homework tasks)	23	36.5
Online learning will only be used for homework-based tasks	8	12.7
We are not putting in any new strategies for online learning in the future	2	3.2
Other	1	1.6

Note: Percentage totals may not equal 100% because of rounding.

When cross-tabulated with the schools that did not have any online provision before COVID-19 restrictions were introduced, 19 out of the 21 schools (90.5%) will be implementing some sort of online learning in the future, with all these schools fully embedding it or using it for a range of subjects both in class and for homework tasks. This important finding suggests that some school leaders are beginning to imagine future education differently, with many moving towards a more strategic blended learning approach for the teaching and learning provision in their schools. Although a chi-squared test of independence showed that there was no statistically significant association between the online provision before COVID-19 restrictions and the strategies for online learning in the future $\left(\chi^{2}(4,n=62)=5.80,\;\;P=0.215\right)$ , the strength of association between these two variables was very strong (Cramer's *V* = 0.31)

From the interview data, although almost all participants agreed that it would play some role, some headteachers were quite circumspect about what role online learning might take, linked to communicating with parents and exam support:

I think it will have a role to play, but to what extent I don’t know yet. I see there are benefits for online learning, particularly for additional support and GCSE revision and support. It's a useful tool for parent communication and giving feedback to parents. Since the second lockdown we’ve been providing parents with weekly feedback.

However, many others were much more enthusiastic about the future:

It will definitely be used for networking, employer engagement, co-delivery, communication, collaborating with other schools and stakeholders. It has made us more efficient and responsive in many ways by being able to hop onto teams and have a discussion with a school 30 miles away.

We’re investigating the skills, talents, experiences and stories that parents have that could be brought into the classroom and shared with pupils. We want to continue building opportunities for parents and pupils to work together and for the school to support parents in their development. We will continue to survey and ensure we’re providing what is of interest.

And some were even reimagining their whole curriculum design:

Online learning will be used in many ways, primarily by personalising provision, reimagining schooling. Moving away from one size fits all tyranny and the unit of 30 in a class. We can redesign the whole organisation. Covid has shone a light on schools and shown that they work for those that schools work for, but they don’t work for others who either can’t cope or agree to comply and fall out of the system.

## Discussion

The findings from our study show that key challenges that were experienced during the period of restrictions were linked to staff expertise and knowledge, with specific issues related to equipment and infrastructure and having the appropriate knowledge and skills to support children from different backgrounds, especially those with SEND, with EAL or who are on FSMs. These findings are in line with emerging work in this area (Bond, 2020; Reimers and Schleicher, 2020) and suggest that targeted and appropriate staff development is crucial moving forward.

Opportunities were linked to more stakeholder engagement and collaboration, and the development of innovative solutions to support and engage children with their online learning experiences during the restrictions. These practices involved including children and parents in key curriculum delivery decisions and the use of collaborative online platforms to develop and share practice and new ideas. Such online staff learning communities have been shown to be very successful in previous studies (Lantz-Andersson et al., 2018; Mostafa, 2021; Sheffield et al., 2018) and suggests a key avenue for future exploration. Innovation in developing online learning during the restrictions has also emerged as a key finding in other studies (Babinčáková and Bernard, 2020; Dwivedi et al., 2020; Oke and Fernandes, 2020; Tamah et al., 2020) and suggests that this practice should be encouraged in the future as schools look to further embed online learning within their curriculums.

**Figure 1.**
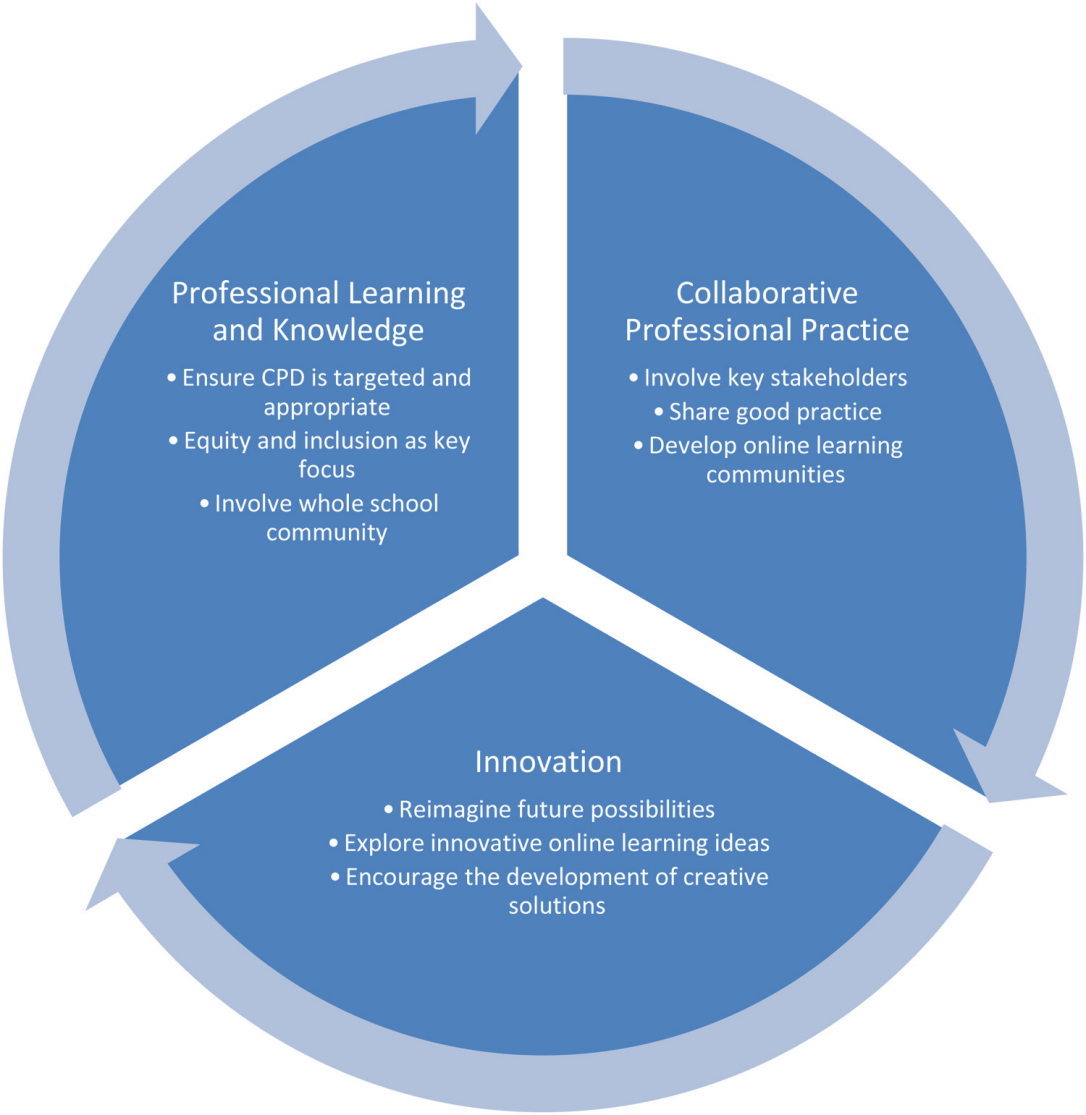
Learning from the pandemic – key principles of successful digital implementation.

Theoretically, extending our previously published work in this area (see Baxter et al., 2023) in which we proposed a model for evaluating how strategically advanced a school or MAT is in relation to digital strategy, in Figure 1, we put forward a conceptual model to help aid understanding of how school leaders can successfully implement digital learning in the future, with a specific focus on staff development and learning. The model illustrates how the key concepts of professional learning and knowledge, collaborative professional practice and innovation interlink and the underlying principles linked to these that emerged from our study. Our model shows that for successful digital implementation to occur, these three concepts need to be considered part of an interlinked whole. For example, concentrating solely on effective professional learning and knowledge is not enough, school leaders also need to focus on how notions of collaborative professional practice and innovation intersect and are supported and developed within the organisation. In this way, the complementarity of the three elements provides a robust base for the successful implementation of digital learning, thus contributing to the literature in this area.

In relation to professional learning and knowledge, our findings suggest that any CPD activities should be targeted and appropriate alongside ensuring that equity and inclusion is a key focus. All such development work should also involve the whole school community. With regard to collaborative professional practice, our findings suggest that a culture of sharing good practice should be encouraged, with all key stakeholders involved in the process. The use of online learning communities should also be supported. Key principles linked to innovation include reimagining future possibilities differently, positively encouraging the development of creative ideas throughout the whole school community and ensuring that all innovative ideas are supported and explored.

In terms of the practical implications of our work, it was clear that many of the schools in this study were keen to fully embrace online learning in the future, but how can headteachers be supported in this endeavour? A key outcome of this study was the development of an online resource aimed at helping school leaders to develop their future online learning strategy and support staff to develop successful learning models within their schools, argued as essential elements of transformative practice in online education (Napal et al., 2020; Vermeulen et al., 2015). The course was developed from findings and insights from the project reported above and elsewhere (Baxter et al., 2023) and is based on the core foundations of equity and inclusion, developing digital capabilities, pedagogical innovation and collaboration and partnerships, as foregrounded and developed in this article and highlighted in Figure 1. The course can be accessed here: *Strategic planning for online learning: A whole school approach.*

## Conclusions

The aim of this article was to explore school leaders’ perspectives and experiences of the challenges and opportunities of leading online learning during COVID-19 restrictions and beyond and importantly identify what lessons can be learned for the future. Using a framework based on factors that have emerged from previous work in this area, we have shown how the key concepts of professional learning and knowledge, collaborative professional practice, and innovation interlink in this context, and the key underlying principles of successful digital implementation that emerged from our study, which we have utilised to develop an online course to help school leaders achieve this aim moving forward. Whilst we believe this conceptual framework has been a beneficial heuristic device for our work, there are of course other factors that could have been included and explored such as headteachers’ leadership behaviours and staff resilience. We suggest that these and other possibilities should be reflected upon in any future research in this area.

There are of course other limitations of our study, with the main one being the low survey response rate which does not allow us to generalise our results in any meaningful statistical way. We also only collected data from school leaders and not teaching staff, pupils or parents which would have given more insight into the whole school community experiences of these issues. Notwithstanding these limitations, it is hoped that the conceptual model and course that was developed out of our work will provide a suitable base for headteachers to develop a successful and sustainable online learning strategy for their schools, with the aim of improving learning and attainment for all pupils. Future planned work will evaluate the success of this online resource, together with measuring the impact on policy and practice.

## Appendix A – Interview Schedule

Before Covid

- Can we start by you telling me about your role and how long you’ve been at the school?
- How many pupils are on roll (academy trusts – how many schools are there in the MAT)
- Before covid, how did you plan and deliver online learning within the school /MAT ?
- Was online learning part of your strategic planning (can we see the strategic plan)
- Before covid who was responsible for developing online learning?
- What percentage of the curriculum would you say was taught online ?
- Do certain subjects use it more than others ?
- Before covid how did you use online learning to include SEND learners?
- What were the main challenges encountered re online learning before covidAnd opportunities?
- What support did you and your teachers have in terms of online learning provision ?

During Covid

- How did the first lockdown affect teaching and learning in your school/MAT ?
- Who was responsible for putting learning online during lockdownAnd now ?
- What role do head of departments play in providing learning for learners during lockdown ?
- What support did you find most valuable during lockdown(government etc)
- What changes or amendments to online learning strategy have you implemented due to Covid19?
- Why did you implement the online strategies that you did?
- What guidance did you have in developing online learning during covidAnd from who ?
- Did your strategic planning include online learning during emergencies such as covid ?
- Did your school planning procedures change due to covid? If so, what were the changes? Can we see your updated strategic plan?
- What were the key challenges in terms of provision, that you encountered during covid? And opportunities?
- How did you deal with them ?
- How did you engage hard to reach learners, e.g. SEND pupils during covid ?
- What strategies are in place for pupils who lack IT kit or have no internet access or limited study space?

After Covid

- What did you learn re online learning during Covid ?
- Has covid changed the way you plan learning, and if so how?
- What role do you think online learning will now play in your school? And in the future of secondary ed?
- What strategies are you putting in place for online learning in the future, both as a response to ongoing pandemic situations and as part of ‘business as usual’?
